# Bilateral pheochromocytomas: clinical presentation and morbidity rate related to surgery technique and genetic status

**DOI:** 10.1530/EC-23-0466

**Published:** 2024-03-01

**Authors:** Sofia Maria Lider Burciulescu, Monica Livia Gheorghiu, Andrei Muresan, Iuliana Gherlan, Attila Patocs, Corin Badiu

**Affiliations:** 1 University of Medicine and Pharmacy Carol Davila Bucharest, Bucharest, Romania; 2 National Institute of Endocrinology CI Parhon, Bucharest, Romania; 3 Department of Laboratory Medicine and Molecular Genetics, Clinical Genetics and Endocrinology Laboratory, Semmelweis University National Institute of Oncology, Budapest, Hungary

**Keywords:** pheochromocytoma, bilateral, adrenalectomy, cortical-sparing surgery, RET mutation, VHL mutation, MEN 2 syndrome, adrenal insufficiency

## Abstract

**Background:**

Pheochromocytomas (PHEOs) are rare catecholamine-secreting adrenal tumors. Approximately 60–90% of bilateral PHEOs are hereditary. We retrospectively analyzed the clinical characteristics of patients with bilateral PHEOs and the morbidity rate (malignancy, tumor recurrence and adrenal insufficiency (AI) rate) related to surgery technique and genetic status of the patients.

**Results:**

Fourteen patients (12.5%, nine women, five men) had synchronous or metachronous bilateral PHEOs (out of 112 PHEO patients who underwent surgery between 1976 and 2021). The median age at diagnosis was 32 years (9–76) (three were children). Nine patients (64.2%) presented synchronous bilateral tumors, five (35.7%) contralateral metachronous tumors, 2–12 years after the first surgical intervention; three (21.4%) were metastatic. Median follow-up: 5 years (1–41), IQR 19 months. A total of 78.5% had a germline mutation (eight RET gene with MEN2A syndrome, three VHL syndrome, three not tested). Post-surgery recurrence was noted in 16.6% of patients (one with MEN2A syndrome and metastatic PHEOs, one with VHL syndrome), with similar rates after total adrenalectomy or cortical-sparing adrenal surgery. AI was avoided in 40% after cortical-sparing surgery.

**Conclusion:**

Bilateral PHEOs are usually associated with genetic syndromes. The surgical technique for patients with hereditary bilateral PHEOs should be chosen based on a personalized approach, as they are at higher risk for developing new adrenal tumors requiring additional surgeries.

## Introduction

Pheochromocytomas (PHEOs) are rare tumors originating from the chromaffin tissue of the adrenal medulla. They secrete catecholamines in excess, generating hypertension paroxysms, the so-called classic triad (headache, profuse sweating, palpitations), life-threatening arrhythmia, stroke, or even death (1). Most PHEOs are rare sporadic unilateral adrenal tumors. However, 25–45% are hereditary (2). Bilateral PHEOs are very rare (7–10% of PHEOs); between 60% and 90% of the patients with bilateral tumors harbor a germline mutation (3, 4, 5). In the last decades, a significant proportion of PHEOs are detected as incidentalomas, and may present with a mild or absent symptomatology (6).

Bilateral PHEOs were most frequently described in the syndromes of multiple endocrine neoplasia (MEN) type 2A and type 2B, in certain families with von Hippel–Lindau disease (*VHL*) or in patients with *MAX* and *TMEM127* gene mutation (3, 7). Not all bilateral PHEOs are components of syndromes. Sometimes bilateral tumors appear in apparently sporadic cases.

Adrenal PHEOs are generally removed laparoscopically when safe and feasible, although the surgeon must prepare to convert to open surgery in some special situations: intraoperative bleeding (PHEOs being highly vascular tumors), capsular injury (risk of hypertensive crisis) or in case of large tumor (>6 cm) or local invasion of the PHEO (8).

Usually, adrenal surgery is associated with low rates of mortality and morbidity, ranging from 6% to 30% (8, 9). The morbidity rate of the bilateral PHEOs surgery must include the evaluation of tumor recurrence, malignancy rate and adrenal insufficiency (AI) (9, 10). Mortality is lower in MEN2 or *VHL* mutation carriers who eventually develop bilateral tumors but are followed regularly (10, 11). The most frequently surgery technique used by surgeon is total adrenalectomy. Many patients develop the second pheochromocytoma several years after the first unilateral tumor was removed, on the same side or contralateral. In hereditary PHEOs a second tumor may occur in the opposite gland, leaving few options for preserving the adrenal function (10, 12).

Based mainly on retrospective studies, cortical-sparing adrenal surgery (CSS) can reduce the risk of AI and has a low risk for recurrence in hereditary PHEOs (3, 8, 12). CSS is mostly recommended in patients with (i) bilateral PHEOs, (ii) tumor size <5 cm, or (iii) in case of a mutation that leads to high risk of metachronous/recurrent PHEO (3, 10). CSS is followed by a low risk of tumor recurrence after 10 years of follow-up (<5–13%) and normal adrenal function in more than 50% of the cases, while total adrenalectomy increases the rate of AI but decreases the risk of recurrence (7, 10). In cases of incidentalomas with low or mild symptomatic presentation, adrenal sparing surgery can be a choice in bilateral form (6).

Our study aimed to describe the clinical characteristics of a series of patients with bilateral PHEOs and to evaluate the morbidity rate (malignancy, tumor recurrence, and AI rate) reported to surgery technique and genetic status of those patients.

## Materials and methods

Following approval by the Ethics Committee of C. I. Parhon National Institute of Endocrinology (no. 4/2020), we retrospectively analyzed records of 112 patients diagnosed with pheochromocytoma who underwent surgery between 1976 and 2021. Diagnosis of PHEO was confirmed after histological analysis for all patients. We collected data on demographics, tumor localization, method of discovery, genetic status, secretion pattern, tumor dimensions, malignancy status, surgery technique, follow-up duration, recurrence status, presence or absence of AI at a maximum 1 month post surgery and outcome. According to tumor localization, we selected 14 patients with bilateral PHEOs, either synchronous or metachronous tumors. Synchronous/metachronous tumors were those diagnosed on the contralateral adrenal gland within 6 months and more than 6 months after initial diagnosis, respectively. The reappearance of PHEO in the same adrenal bed after complete/cortical-sparing resection was defined as recurrence. A noradrenergic secretion pattern was defined as predominant increases of normetanephrines (NMN), while metanephrines (MN) concentrations were <2× the upper limit of normal (ULN), whereas an adrenergic secretion pattern was defined as increments >2× ULN for both metabolites. We used plasma catecholamine metabolites detection method, as mentioned in a previous paper by Stefanescu *et al.* (13).

Imaging diagnosis was made using contrast tomodensitometry (CT scan), in all patients, as this method is elective in this disease. Metastasis were also diagnosed using CT scan (MIBG/PET-CT not available at the time of diagnosis).

Metastatic disease was defined as the presence of PHEO in a nonchromaffin-containing tissue site, such as lymph nodes, bone, liver, and lung. Imaging techniques used for primary tumors and metastatic disease were CT scans and MRIs.

The presence of AI was defined as low morning (08:00–09.00 h) cortisol level (<3 µg/dL) and insufficient cortisol response to a stimulation (Synachten) test at a maximum 1 month post surgery and the need of gluco- and mineralocorticoid lifelong replacement therapy.

### Genetics

The DNA extracted from patients included in this study was stored in the BioBank of C. I. Parhon National Institute of Endocrinology, based on their informed consent. Currently in our center only *RET* gene testing is available. Thus, 8 patients underwent direct analysis (Sanger sequencing) of RET proto-oncogene, based on their clinical phenotype and family history for patients with MEN2A. Oligonucleotide primers for the amplification of different *RET* exons were designed at intronic sequences flanking exons 8, 10, 11, 13, 14, 15, and 16. PCRs were performed in a final volume of 25 µL containing 20 mM Tris–HCl (pH 8.4), 50 mM KCl, 1.5 mM MgCl_2_, 0.2 mM deoxynucleotide triphosphate, 1 U Taq polymerase, 1 mM specific primers and using 100 or 200 ng of genomic DNA as input. Sanger sequencing for RET gene was available in our center starting in 2018.

For three patients with VHL, the mutation was confirmed using next-generation sequencing (NGS) based technique, in Hungary using a Commercial Cancer Panel Trusight Hereditary Cancer Panel from Ilumina® targeting 113 genes. In this panel genes related to PPGLs (*VHL, SDHA, SDHB, SDHC, SDHD,SDHAF2, NF1, MAX, TMEM127, FH, CDKN1B, MET, PPKAR1A*, and other non-PPGL-related genes) were covered.

For the rest of three patients, the DNA samples were not available: one patient was lost of follow-up in 2005, before the availability of genetic tests and in the other two patients, with metastatic disease, we had no time for DNA extraction (one patient lost to follow-up after 1 year, and another one died).

All statistical procedures were performed using SPSS 23.0 software package (SPSS Inc.). Continuous variables were described in terms of mean ± s.d. if their distribution was normal according to the Kolmogorov–Smirnov test and in terms of median and range/IQR (interquartile range, otherwise). For statistical analysis, we used non-parametric correlations, Kruskal–Wallis test, Fisher test, and Student’s *t*-test where appropriate. A two-sided *P*-value <0.05 was considered to indicate statistical significance.

## Results

### Demographics

Our cohort was selected from a series of 112 patients with PHEO with a mean age at diagnosis 49.8 ± 15 years, 98 (87.5%) presenting unilateral tumor, removed by total adrenalectomy; the median follow-up duration was 5 years, IQR 18 months. The median age of diagnosis in patients with unilateral PHEOs, was significantly higher than in those with bilateral PHEOs (49 (19–81) vs 32 (9–76), *P* = 0.009). Most of the patients were women in both groups: 66.7% in unilateral and 67.7% in bilateral group respectively.

The study group included 14 patients with bilateral PHEOs (Table 1) (12.5% out of 112 patients): nine women, five men, three of them (21.4%) were children at diagnosis. Nine (64.2%) presented synchronous bilateral tumors, whereas five patients (35.7%) presented contralateral metachronous tumors, 2–12 years after the first surgical intervention. The median follow-up duration for these patients was 5 years (1–41), IQR 19 months. In patients with unilateral tumors, only 26.9% of them had hereditary disease, while in bilateral group 78.5% of patients had a germline mutation.

**Table 1:** Characteristics of patients with bilateral PHEOs.

Case	Age, gender	Genetics	Clinical aspects	Metastatic/benign	Maximum tumor diameter (mm)	MN /NMN (ng/L)	Type of Surgery	AI	Follow-up up period (years)	Recurrence time/metachronous Disease (yrs)	Outcomes
1	14, female	VHL c.245G>T, p.Arg82Leu	S. Hemangioblastomas	Bg	NA	30/1524	O+L (CSS, then unilateral T for recurrence)	No	38	R 7 (post CSS) R 24 (post T)	Exitus
2	14, female	VHL c.245G>T, p.Arg82Leu	S. Hemangioblastomas	Bg	62	105/2379	L (CSS)	Yes	4	–	Active follow-up
3	9, female	VHL c.482G>A, p.Arg161Glu	S. No syndromic tumors	Bg	37	69/3270	L (CSS)	Yes	3	–	Active follow-up
4	22, female	RET c.1900T>C, p.Cys634Arg	S. MTC	Bg	67	2038/1390	L (CSS+T)	Yes	5	–	Active follow-up
5	51, male	RET c.1900T>C, p.Cys634Arg	S. MTC, PHPTH	Bg	76	1015/2545	L (T)	Yes	3	–	Active follow-up
6	25, male	RET c.1900T>C, p.Cys634Arg	M. MTC, PHPTH	Bg	30	1161/1090	O+L (T)	Yes	19	12	Active follow-up
7	40, male	RET c.1902C>G, p.Cys634Trp	S. MTC	Mt Renal	60	164/1167	O (T, unilateral)	No	41	R 11	Active follow-up
8	50, female	RET c.1852T>C, p.Cys618Arg	S. MTC, PHPTH	Bg	60	499/1120	L (Right, then left-T)	Yes	2	–	Active follow-up
9	22, female	RET c.1900T>C, p.Cys634Arg	M. MTC, PHPTH	Bg	70	877/7720	L (T)	Yes	14	2	Active follow-up
10	32, female	RET c.1902C>G, p.Cys634Trp	M. MTC	Bg	50	120/173	L (T)	Yes	22	5	Active follow-up
11	40, female	RET c.1902C>G, p.Cys634Trp	M. MTC	Bg	73	1560/1380	O+L (T)	Yes	25	9	Active follow-up
12	60, male	–	M.	Bg	15	NA	O (CSS)	No	10	7	Active follow-up
13	36, male	–	S.	Mt Retroperitoneal lymph nodes, lungs, liver, bones	NA	127/5627	NA	NA	3	–	Exitus after surgery
14	76, female	–	S.	M Liver	36	NA	NA	NA	1	–	Lost to follow-up before surgery

AI, adrenal insufficiency; Bg, benign; CSS, cortical-sparing adrenal surgery; L, laparoscopic surgery; M., metachronous; MN = plasma free metanephrines (*n* = 10–90 pg/mL); Mt, metastatic; MTC, medullary thyroid carcinoma; NA, not available; NMN, plasma free normetanephrines (*n* = 20–200 pg/mL); O, open surgery; PHPTH, primary hyperparathyroidism; R, tumor recurrence; S., synchronous; T, total adrenalectomy.

### Clinical presentation

Four patients (28.5% of the bilateral PHEO) were diagnosed through genetic screening, on a regular follow-up basis. The rest of 10 (71.4%), were symptom-based diagnosed: classical clinical triad, hypertension paroxysms, intense catabolic activity symptoms (fatigability, loss of weight), abdominal pain were described. One pediatric patient was diagnosed after an epileptic episode.

### Genetics

In 11 patients (78.5%) we identified a germline mutation; eight patients presented MEN2A syndrome (seven with 634 codon mutation and one with 618 codon mutation), while three patients had *VHL* syndrome.

For the rest of 3, we could not perform the genetic screening due to the time of diagnosis (before the genetic screening facilities available). They were considered ‘unknown’ PHEOs (non-syndromic, not genetically tested).

Patients with bilateral PHEOs and *RET* mutations represent 44.4% of all patients with RET mutation identified in our series of 112 patients. No *TMEM127, MAX or SDHx* mutations were detected in our screened cases.

### Secretion pattern and tumor dimensions

A noradrenergic secretion pattern was present in five patients with bilateral PHEOs, while seven patients presented with an adrenergic pattern. Preoperative hormonal data were not available in the other two patients. The adrenergic pattern was specific for patients with MEN2A syndrome: six out of eight patients with RET mutation had an adrenergic pattern (Table 1).

Furthermore, urinary and plasma NMN levels were non-significantly higher in patients with bilateral tumors than in those with unilateral PHEOs (*P* = 0.76 for urine NMN and 0.29 for plasma NMN). Tumor dimensions in patients with bilateral PHEO were non-significantly higher in those with syndromes (maximal diameter mean = 50.7 ± 16 mm) compared to unknown cases (mean = 50.6 ± 21 mm); *P* = 0.99.

### Morbidity rate

#### Malignancy

Three out of 14 patients (21.4%) presented metastatic PHEO (two of them at the time of diagnosis). Sites of metastases were: kidney, lung, liver, vertebral, periaortic lymph nodes, retrocaval lymph nodes and lymph nodes adjacent to the adrenal gland. One of these three patients with metastatic PHEO had RET mutation, and the other two were ‘unknown’ cases (Table 1).

#### Morbidity rate related to surgery technique

- Five patients with bilateral PHEOs had *CSS*: 4 bilateral, 1 CSS on one side and total adrenalectomy on the other side (case 4). AI was recorded in three (60%) of them. High tumor dimensions, or low vascularization of the remnant adrenal gland could be some factors which conducted to AI. Recurrence was documented in one patient with VHL syndrome and benign bilateral PHEOs at 7 years (bilateral recurrence after CSS) and 24 years from the first surgery (unilateral recurrence after total adrenalectomy), respectively (case 1).
- Seven patients had total adrenalectomy. AI developed in all patients after bilateral total adrenalectomy (100%). Recurrence was detected in one patient with MEN2A syndrome who had recurrence at 11 years and a calcified contralateral tumor that was inoperable because of the calcification. The patient developed metastatic disease (case 7).
- In two patients we had no data regarding the surgical technique: one patient with metastatic PHEOs died after surgery and no information about the time or cause of death was available; another patient was lost to follow-up before surgery (cases 13, 14).

Overall, in our cohort we found recurrences in 2 out of 12 patients (16.6%). The recurrence rate reported to surgery technique was:

- for patients with CSS (including the patient with CSS + T) – 1/5 patients (20%).
- for patients with total adrenalectomy (including patient with CSS + T and the patient with bilateral recurrence) – 2/9 (22%), *P* = NS compared to CSS. Surgeon experience, tumor dimensions, vascularization of the remnant adrenal and genetic status as well as the co-occurrence of multiple tumors in the spared adrenal are other important factors that influence the post-surgery outcome.

#### Morbidity rate related to genetic status

Two out of 11 (18.2%) of patients with genetic syndromes developed recurrence and 5/11 (45.45%) patients with genetic syndromes had metachronous tumors at 2–24 years of follow-up (Table 2).

**Table 2:** Tumoral outcome related to genetics in patients with bilateral PHEOs.

Genetic background	Synchronous	Metachronous	Recurrence	Metastatic	Observation
MEN2A (*n* = 8)	4	4	1 (total adrenalectomy)	1	The patient with recurrent disease had metastatic PHEO
VHL (*n* = 3)	3	–	1 (cortical sparing + total adrenalectomy)	0	The patient developed recurrences after CSS at 7 years, then after T adrenalectomy at 24 years
‘Unknown’ (*n* = 3)	2	1	0/1	2	Metastatic PHEOs (in two patients) were bilateral at diagnostic
TOTAL	9/14	5/14	2/12	3/14	

Out of eight patients with MEN2A Syndrome and bilateral PHEOs, four presented with synchronous tumors and 4 with metachronous tumors (Table 2).

Only one MEN2A patient had one recurrence at 11 years – after total unilateral adrenalectomy. This patient also presented medullary thyroid carcinoma with metastatic disease, calcification of the contralateral adrenal gland (that made it inoperable) and metastatic PHEO (case 7).

Seven out of eight patients with MEN2A syndrome developed AI – six of them after total adrenalectomy and one after cortical-sparing (one side) and total adrenalectomy (on the other side). The only MEN2A patient without AI and with metachronous tumor was the case presented above (case 7).

Out of three patients with VHL syndrome, all had synchronous tumors and all had cortical-sparing surgery. One patient had recurrences at 7 and 24 years (case 1). None of the VHL patients developed metastatic disease (Table 2). Two out of three VHL patients developed AI despite CSS surgery. Of three patients without documented mutations, two had synchronous tumors (both metastatic, inoperable) and 1 patient had metachronous tumor at 7 years – he underwent cortical-sparing surgery, without recurrence (Table 2).

#### Outcome at the last follow-up

Two of 14 patients (15.3%) died: one man, aged 36 died due to metastatic disease although the adrenalectomy was performed, another one with benign PHEO but with VHL syndrome died due to other syndromic tumors (medullar and cerebral tumors).

One patient was lost to follow-up before surgery. One patient with RET mutation has an active metastatic disease (MTC and pheochromocytoma), being under treatment with tyrosine kinase inhibitors; other two patients with RET mutation developed recurrent MTC but no recurrence of PHEO. The rest of the patients were disease free at the last follow-up.

## Discussion

Our study summarized clinical characteristics, morbidity rate, and outcome reported for the surgical technique and for the genetic status of 14 patients with bilateral PHEOs diagnosed and treated in a referral center of endocrinology from Romania.

### Bilaterality and malignancy

The percentage of bilateral disease found in our cohort (12.5%) is similar to that in the literature (4, 10, 11). The median age at diagnosis of patients with bilateral tumors was lower than in patients with unilateral PHEOs, a fact that is justifiable by the genetic syndromes associated with bilateral tumors and is consistent with other studies (3, 14). More than half of patients (9/14) presented with synchronous bilateral disease, a finding that was expected based on the previously described cases, where the metachronous disease was present in up to 30–50%, while synchronous tumors were present in more than 50% of the cases (3, 4, 15). However, the presence of unilateral tumor does not exclude the possibility of developing contralateral tumor, many years after the first surgery, as happened in our cohort – the shortest and the longest interval until a second contralateral tumor appeared were 2 years and 12 years respectively. Thus, long-life follow-up is a reasonable approach for patients with PHEOs, especially in those with genetic syndromes.

The malignancy rate in our study group was 3/14 = 21.4% – higher than 10% described previously (6, 10, 15). All patients had bilateral tumors at diagnosis, variable profile of catecholamine secretion (adrenergic/noradrenergic/NA), but small primary tumors (≤4 cm); in two cases metastasis were present at diagnosis, in one case metastasis developed on follow-up (at 11 years). Such high malignancy rate was previously described only in pediatric patients (11, 16). In our cohort, all three patients were adults at the time of diagnosis. One patient had RET mutation – in the other two cases the genetic evaluation was not available; extended germline mutations in genes associated with PHEO development and a somatic genetic test would elucidate and extend the knowledge about genotype of patients with bilateral and metastatic disease.

Particularly, in case of a metastatic PHEO, a multidisciplinary approach is essential. A board meeting between endocrinologist, radiologist, surgeon, histopathologist, oncologist, and radiotherapist should be the central part in the management of a patient with metastatic PHEO. Moreover, as we live in an informatized era, with the help of artificial intelligence, we can develop new methods for the prediction of metastatic/recurrent disease, corroborating all the aspect identified by each specialist. Radiomics, using texture CT aspects, is a new direction for predicting a higher probability for metastatic disease (17).

### Genetics

Genetic syndromes were detected in 78.5% of this cohort. Our percentage corresponds to that found in other studies, were bilateral PHEOs were hereditary in 60–90% of cases (3, 4, 5, 18, 19).

Our results demonstrated that patients with bilateral PHEOs most frequently harbor *RET* 634 codon pathogenic variant and *VHL* mutation. The approximate frequency of pheochromocytoma (reported in the literature) in these syndromes is 10–20% in *VHL* syndrome, 50% in MEN2 (3, 4, 17, 20). In our large series of 112 patients, bilateral PHEOs and *RET* mutations represented 44.4% of all patients with *RET* mutations. These two genes were described to be most frequently involved in bilateral PHEOs, but with a predominance of synchronous tumor in *VHL* syndrome, as reported in our cohort also (15), while in another large cohort patients with MEN2A syndrome presented more frequently with synchronous tumors than patients with *VHL* (67% vs 57%) (3).

Notably, patients with *RET 634* codon mutation were most prevalent in our cohort, a fact that was also described in other studies (3, 21, 22). In three patients who had no syndromic features the genetic evaluation was not available; as two out of cases were metastatic, a germline or somatic mutational base may be presumed.

### Tumor dimensions and hormonal profile

Patients with *VHL* mutation and sporadic cases secreted predominantly norepinephrine, while those with *RET* mutation had adrenergic secretion pattern – an expected pattern – based on phenotype–genotype correlation described in the algorithm for genetic testing in Endocrine Society guidelines (7, 22, 23). Tumor dimensions were non-significantly higher in patients with germinal mutation compared to sporadic cases. This finding suggests that patients with bilateral tumors do not have a higher development rate than those with unilateral tumors.

### Surgical interventions and recurrence risk

Bilateral cortical-sparing adrenalectomy became an option for patients with bilateral PHEOs since it was first described by Walz in 1996 (24). However, due to a higher risk of recurrence in the older studies, it has been used mostly in patients with hereditary PHEOs in whom the prevalence of bilateral tumors is high and the risk of malignancy is reasonably low (25). In our cohort, most of the patients were operated on using the total adrenalectomy technique (64.2%).

Overall, we had a recurrence rate (16.6%) similar with other studies, where the recurrence rate was 3–13% (3, 26, 27, 28). When analyzing patients with cortical-sparing surgery, we observed that 20% (*n* = 1/5) of patients with CSS developed recurrence, probably due to the genetic background (VHL mutation). Two recent studies, one a multicenter international study (including 625 patients with bilateral PHEOs) and another one on 170 patients with either bilateral and unilateral PHEO, reported a lower recurrence rate of 13% after CSS, mostly in patients with *VHL* and MEN 2 (3, 29). Another multicenter study with 82 cortical-sparing surgeries in bilateral MEN2A PHEOs showed a 5% recurrence rate (19). The risk of recurrence was similar after CSS compared to total adrenalectomy in bilateral PHEOs in some series (<5% in (19)) but higher (OR 3.72, *P* = 0.003) in a recent meta-analysis of 985 cases (27) and in a large cohort study of 625 cases (13% vs 0.66% after follow-up of 4–17 years (3)).

The recurrence rate in our patients with total adrenalectomy was similar to those with CSS – 22% (2/9 cases), but these percentages may be biased by the low cohort number. These results lead us to the conclusion that surgical technique per se is not the only predictor factor for tumor recurrence. Surgeon experience, tumor dimensions, vascularization of the remnant adrenal and genetic status as well as the co-occurrence of multiple tumors in the spared adrenal are other important factors that influence the post-surgery outcome (12, 28). On the other hand, when reported to genetic status, our findings correspond to other results from the literature where the recurrence rate in patients with RET and VHL pathogenic variant, treated with CSS was between 5% and 38.5% (3, 10, 21, 26).

### Type of adrenal surgery and adrenal insufficiency

As expected, all the patients undergoing bilateral total adrenalectomy developed AI. In contrast, only 60% of patients with cortical-sparing adrenalectomy developed AI, requiring lifelong steroid replacement (mean dose 15 mg HC/day). Similar rates of AI after CSS (43%) in patients with bilateral PHEOs have been reported in a series of 82 MEN2 (18) and in a recent meta-analysis of more than 1400 patients, which showed that patients with CSS had a three times lower risk of developing acute adrenal insufficiency or steroid dependency, compared to those with total adrenalectomy (27). In patients with CSS, AI may occur due to insufficient blood supply to the remaining adrenal cortex, insufficient cortical tissue or in case of pre-surgery mild autonomous cortisol secretion (MACS), especially in patients with MEN2A syndrome, where usually PHEO is preceded by adrenal hyperplasia (9, 28, 29, 30).

The time of follow-up after bilateral adrenalectomy with at least one CSS in order to detect the need for glucocorticoid replacement is not well established.

These patients should receive hydrocortisone (50 mg i.v. every 8 h) during the immediate postoperative period, followed by transition to oral hormone replacement with hydrocortisone (20–30 mg/day) and, when needed, fludrocortisone (0.1 mg/d) (9, 25, 27, 28).

One approach would be to evaluate adrenal function with short Synacthen test before glucocorticoid intake at 1-week post-surgery. If the patient developed AI, then periodical measuring of morning plasma ACTH and cortisol before glucocorticoid intake (e.g. at 1, 3, 6, and 12 months) and clinical evaluation should guide the clinician to the adequate substitution dose (20, 30, 31).

### Survival

Two of our patients died (15.3%). In case 1, the patient died at 46 years old, of causes unrelated to PHEOs but due to other VHL-associated tumors (recurrent cerebral and spinal cord tumors but not renal cell carcinoma). It is known that the frequency of deaths caused by other tumors especially renal cell carcinoma in VHL at the age 40–52 is approximately 78% (2, 32, 33). The other patient died at the age of 37 due to advanced metastatic disease at the time of presentation. Role of other PHEO-associated gene, especial SDHB, cannot be excluded in this patient, but due to the lack of DNA material we could not confirm this hypothesis.

In a recent meta-analysis, CSS was not associated with decreased survival compared with total adrenalectomy in bilateral PHEOs at a follow-up of 4.9 to 13 years (27). Mortality was mostly associated with malignancy or morbidity unrelated to pheochromocytoma (3).

The main limitation of the study is that only 3 out of 14 patients were fully genetically tested. Other limitations of our study include the retrospective design and since PHEO is a rare disease, a limited number of the cohort. The patients were selected consecutively. We also had restricted access to perform genetic testing. Patients in our cohort were retrieved from an extensive period of time. The surgical approach was decided onsite by surgeons depending on personalized criteria, according to their experience and to the surgery technique applied in each period of time in which surgery was performed.

## Conclusion

Our study ascertained that bilateral PHEOs are usually associated with genetically determined syndromes, most frequently with VHL and RET mutation, but they also can be identified in sporadic cases. We identified a higher rate of malignancy in bilateral PHEOs compared to overall population of PHEOs. Cortical-sparing adrenalectomy is often ideal for individuals with hereditary pheochromocytoma as they are at higher risk for developing metachronous tumors that would require additional surgeries. Therefore, a genetic test is essential for patients with PHEOs.

The surgical technique used for each patient, including cortical-sparing surgery, does not guarantee a normal adrenal function after surgery, as well as total adrenalectomy does not predict a recurrence-free evolution. Before choosing the surgery technique, there are some factors that should be evaluated by a highly specialized multidisciplinary team including the presence of a mutation predisposing to an increased risk of future tumor recurrence or malignancy, tumor size and adequate vascular supply to the remaining adrenal tissue. The medical team should always balance the risk and benefits of the surgical approach. AI as well as recurrence/metastatic disease are two outcomes with a bad prognosis and low quality of life that lower the life expectancy. Therefore, patients with bilateral PHEOs require long-term follow-up with clinical evaluation and biochemical testing. They should be thoroughly instructed about adrenal crisis and optimization of the glucocorticoid and mineralocorticoid substitution in order to minimize the morbidity and mortality associated with their disease.

## Declaration of interest

The authors declare no conflict of interest that could be perceived as prejudicing the impartiality of the study reported.

## Funding

Publication of this paper was supported by the University of Medicine and Pharmacy Carol Davila, through the institutional program Publish not Perish.

## Author contribution statement

Conceptualization, SMLB, MLG, CB; methodology, SMLB, AM, AP; formal analysis SMLB; investigation, SMLB, MLG, CB, AM, IG, AP; writing – original draft preparation, SMLB; writing – review and editing, SMLB, MLG, CB, IG, AP; All authors have read and agreed to the published version of the manuscript.
